# Supplementary material for: The association of motivation with mind wandering in trait and state levels
Source: PLoS One. 2020 Aug 13;15(8):e0237461. doi: 10.1371/journal.pone.0237461 (PMC7425929; doi:10.1371/journal.pone.0237461)
Supplement: Attachment — Submitted filename: response_letter4.docx [file pone.0237461.s001.docx]

Dear editor and reviewers:

Thank you very much for your kind consideration of our manuscript (PONE-D-20-10065), titled “The Association of Motivation with Mind Wandering in Trait and State Levels.” Following your valuable comments, we carefully revised our manuscript, which once again underwent English proofreading. Point-by-point responses are given below in which the replies to the reviewers’ comments are listed first, followed by the editor’s comments. In the main paper, we have highlighted the revised text.

**Reviewer 1**

Major point 1 – non-independence of observations in the data submitted to the regression analysis.

Recommendation: linear mixed model

On Page 12, the authors report: «To investigate which factors had an impact on state MW, we conducted a multiple regression analysis to predict state MW (Probe) based on their trait and state motivation (Inv-AS and IMI), and indices of trait MW (MWQ and DDFS) with a stepwise method...” This regression analysis should be conducted including subject (i.e., participant) as a random effect along with estimation of the fixed effects (i.e., the contribution of the measured variables). Because the design of this subsample is within-subjects (i.e., each participant contributed to all measurements), the data within people are more similar than data between people, and thus the assumption of independence of observations, which is an assumption of linear regression, is not met. Making this explicit in the model is necessary to estimate fixed effects (i.e., the contributions of the trait and state measurements) adequately. E.g., because the contributions of the individuals from different scales are more similar within the individual, the model can overestimate the strength of the statistical relationship, if within- participants’ data dependency is not specified in the model. Accordingly, for this analysis linear mixed effects models are the appropriate tool (Singmann, H., & Kellen, D. (2017). An introduction to mixed models for experimental psychology. New methods in neuroscience and cognitive psychology). Using p-value statistics, this is analysis can be conducted with the free software R using the package “lme4” (Bates, Mächler, Bolker, & Walker, 2015) or package “afex” (Singmann, Bolker, Westfall, & Aust, 2016).

Response: We thank the reviewer for this pertinent suggestion. We would like to discuss it further to refine our paper as we have approached it in a different manner. If the subject effect was a confound, we should have controlled this effect by “partialling out” or considering it as a “random effect” as you suggested. We read the Singmann and Kellen (2017) which is an organized introduction to use mixed models for data analysis in the experimental psychology area. In this paper, they raise an example that “For example, when measuring response latencies a participant that is generally slower than her / his peers will respond comparatively slower across conditions, thus making the data points from this participant correlated and non-independent.” We agree with this as in such analysis, the researcher would like to know the relationship of response time (RT) between 2 different conditions, so the “general response time” would be a strong confounding factor. The effect of the condition would be accounted for by the effect of general RT and, in this sense, we could not say that the RT of condition 1 and 2 are independently measured.

However, in our study, we aimed to investigate the association between the DV (i.e., state MW) and several IVs (i.e., trait MW, trait and state motivation). Apart from the target concepts, which were “motivation” and “MW”, there was no confounding factor which uniformly affected the variables. Since our aim was to investigate this association, we focused on the inter-individual variation, which you indicated as the “random effect.” Moreover, although you mentioned that “the model can overestimate the strength of the statistical relationship, if within- participants’ data dependency is not specified in the model,” here we aimed to indicate that this model did not explain the DV well; thus the focus is not on the weaker statistical relationships.

Major point 2 – The authors conclude that “there is no association between trait motivation and state MW” (page 15). This conclusion may be problematic, seeing as it is based on a statistical null result. Seeing as p-value testing can only reject H1 but not prove H0, this statement could be reformulated. It could also be specified whether the authors conclude that this association does not exist at all or do they wish to express that it does not exist in their data.

Response: We understand that the inferential statistics could not have the ability to prove “no difference.” Although, we drew the conclusion based not only on the statistical result but also on the plot; thus, we could insist that the association between trait motivation and state MW has not been confirmed in the current study. We revised the sentences as:

“Since our data did not satisfy this assumption, in addition to the results of multiple regression analysis, we conclude that, at least from the current dataset, there is no association between trait motivation and state MW.”

Minor point It would be helpful to the reader to provide more detailed descriptions of the questionnaires (i.e., how many items they contain, what the possible range of scores is).

Response: We added this information into the relevant sections, as well as revisions for reviewer 2 as:

“The Japanese version of AS, which is a fourteen item 4-point scale whose scores range from 0 to 42 (e.g., “Are you interested in learning new things?”), was well-known in Japan (Okada, Kobayashi, Aoki, Suyama, & Yamaguchi, 1998).”,

“A difference between the MWQ (five items with a 6-point scale whose scores range from 5 to 30) and DDFS (twelve items with a 5-point scale whose scores range from 0 to 48) is that the…”, and

“The current IMI asks participants to rate their level of motivation for carrying out a given task at hand with nine items consisting of a 7-point scale with scores ranging from 9 to 63 (e.g., “I put a lot of effort into this”).”

2) A possible concern with the Apathy Scale (which could be discussed) is that the scale only goes from 0 to minus X points (as I assume; please see the point above about the interest to describe the scales more thoroughly). I.e., if I correctly understand the scale idea, it is conceived to measure apathy on a continuum from 0 apathy to very high level of apathy. However, if so, this way of measurement does not capture the upper region of motivation, namely, higher motivation than the average. It seems likely, therefore, that the AS scale can capture the area of average to very low motivation, whereas it cannot capture motivation levels that are higher than average. Is this true, and if yes, does this have implications for the power of the data to observe the hypothesized relationship with Mind Wandering?

Response: We thank the reviewer for the pertinent suggestion. We found that the dispersion of AS scores seemed to be smaller in the higher motivation than in the lower motivation population. We added this point into the limitations section as:

“Third, although we have denoted the good dispersion of AS scores, it seems that the AS did not capture the upper level of motivations rigorously (i.e., smaller dispersions in higher motivation). Since this might affect the observation, it is recommended that subsequent studies use a different scale for assessing motivation which could capture a wider range of motivational traits.”

3) On page 12, section “Relationship between trait and state level indices», reports: “We found a correlation between the trait and state level of each motivation index, r = 0.31, p = 0.001. After several times of reading the manuscript, I now assume that this statement refers to “We found significant correlation between the IMI and the AS”. If my understanding is correct, it would be helpful to the reader to reformulate the sentence. At the present formulation it makes the impression that there were more than two indices, and they were correlated multiple times, which is probably not what the authors intended.

Response: We thank the reviewer for this suggestion. We revised this as:

“We found a significant correlation between the trait and state level of motivation indices (i.e., AS and IMI), *r* = 0.31, *p* = 0.001.”

Very small points

1) On Page 3, the authors write «MW is not the umbrella term for the psychological phenomenon to which we refer, including task-unrelated thought, stimulus-independent thought, self-generative thought, and zoning/tuning out.» Considering the context, I understand they wish to say MW IS the umbrella term?

Response: We thank the reviewers for pointing this out. We deleted “not” from that sentence and have rephrased it as follows:

“MW is the umbrella term for the psychological phenomenon to which we refer, including task-unrelated thought, stimulus-independent thought, self-generative thought, and zoning/tuning out.”

2) The publication of Smallwood and Schooler (2006 ,The Restless Mind) is not a study, but a review.

Response: We revised the “studies” to “reviews.”

3) Page 7, «Behavioral Task and Questionnaire for State Level Investigation». Please cite the developers of the SART task (Robertson, I. H., Manly, T., Andrade, J., Baddeley, B. T., & Yiend, J. (1997). Oops!’: Performance correlates of everyday attentional failures in traumatic brain injured and normal subjects. Neuropsychologia, 35(6), 747–758).

Response: We cited that article in the corresponding section.

**Reviewer 2**

1) Building on Seli et al. (2016, 2017) the authors argue that they assess state mind wandering by including thought probes in an ongoing task. I am not completely convinced that this is indeed a pure state measure of mind wandering. In line with my concern, the authors report correlations of .5 between the state and trait mind wandering measures. Seli et al. may provide good arguments for their view but the authors currently do not make them transparent. In order to convince the readers that state and trait mind wandering is dissociable the authors should elaborate their and Seli et al.’s rationale in the Introduction section.

Response: We thank the reviewers for this suggestion; however, could this maybe be made clearer as we are not sure what was meant by this suggestion. Seli et al. (2016) just denote the difference as “… the trait level – when people are reporting on their levels of mind wandering in daily life – and also at the state level – when people respond to thought probes asking them to report on their momentary cognitive experiences in the laboratory” in the Introduction and “Whereas the state-level measures provide construct validity for the trait-level measures, the trait-level measures indicate that the state-level measures are generalizable to everyday situations. This ﬁnding extends and further reﬁnes previous demonstrations of a correspondence between overall mind-wandering rates in the laboratory and in everyday life” in the Discussion section. They primarily focused on the distinction between spontaneous and deliberate MW in that study, so we believe they did not provide a rational explanation for the state measure of mind wandering. As we had defined in the original manuscript, “ ’trait MW’ is defined as how people perceive their level of MW in daily life, while ‘state MW’ is determined by how people respond to thought probes requesting feedback on their momentary psychological experiences in the laboratory,” we think it was sufficient to define the state MW. Moreover, the correlation coefficient of 0.5 between trait and state MW was larger than the report of Seli et al. (2016)’s. However, we added the following sentences to the limitation section:

“Additionally, the SART might not be a pure measurement of state MW; although, state MW was operationally defined by SART in the current study. However, so far at least, the probe caught method is a popular and reliable method to measure MW (Weinstein, 2018).”

2) The authors should provide example items for all questionnaires they used so that the reader can better understand how they assess the constructs of interest.

Response: We had already provided examples of MWQ and DDFS in the “Questionnaires for Trait Level Investigation” section of original manuscript. Examples of the other indices that were added to the Method section are:

“The Japanese version of AS, which is a fourteen item 4-point scale whose scores range from 0 to 42 (e.g., “Are you interested in learning new things?”), was well-known in Japan; although, it was not validated (Okada, Kobayashi, Aoki, Suyama, & Yamaguchi, 1998).”,

“A difference between the MWQ (five items with a 6-point scale whose scores range from 5 to 30) and DDFS (twelve items with a 5-point scale whose scores range from 0 to 48) is that the…”

“The current IMI asks participants to rate their level of motivation for carrying out a given task at hand with nine items consisting of a 7-point scale with scores ranging from 9 to 63 (e.g., “I put a lot of effort into this”).”

3) Relatedly, it would be important to know whether there is a conceptual overlap between apathy and trait mind wandering. That is, are the items used to assess the one construct semantically similar to the items used to assess the other one or not?

Response: We believe there is no overlap between them. The list of AS can be seen [here](http://scielo.isciii.es/scielo.php?script=sci_arttext&pid=S0213-61632006000200005) and the MWQ [here](https://www.ncbi.nlm.nih.gov/pmc/articles/PMC3753539/). We added the following sentence to the Methods section:

“There is no conceptual overlap between the constructs assessed by AS and MWQ/DDFS.”

4) The authors should indicate how they determined the thought probe presentation within the SART. It would be particularly interesting to know whether they occurred primarily after go or after no-go trials.

Response: We revised the corresponding section as:

“The target and the thought probe randomly appeared at the rate of 5% of all trials and of 2.3% (i.e., 15 probes / 650 presentations), respectively”

5) If the thought probes were presented after no-go trials mostly, it might be the case that participants used their SART performance for evaluating whether they had been on-task or off-task. Similarly, it might be that they used their SART performance to evaluate their motivation to perform this task after task completion. For this reason, the authors should also calculate and report SART error rates and correlate them with state mind wandering and state motivation. My question would be whether the correlation between state mind wandering and state motivation is still present when task performance is controlled for.

Response: Although this point is important for the correlation of state MW tendency with other indices, we thought that listing the correlations of behavioral performance may blur the main argument of this study. However, as you indicated, controlling performance would be necessary to claim the significant Motivation-MW association. We calculated it and reported it in the Results section as:

“Moreover, this relationship remained significant when the task performance (i.e., true negative rate) was controlled (*r* = −0.27, *p* = 0.005).”

and in Discussion section as:

“We replicated this state level association independent of task performance and demonstrated that such an association was also present at the trait-level.”

6) It would be really nice if the authors were able to make this data set available. Currently, the authors rather generically state that they refrain from doing so because of ethical issues. But what are the exact ethical concerns preventing the authors from sharing their data publically?

Response: We did not explicitly denote that the raw data will be openly available during informed consent; although, we mentioned “the statistically-processed data will be openly available.” We are currently contacting the ethics committee for the availability.

**Editor**

(1) Please indicate the full contact information of the ethics committee from which the data could be requested. Please also indicate the ethical reasons that prevent public sharing of the data.

Response: As for the 6) in reviewer 2, the ethical reason was that we did not explicitly denote that the data will be openly available in publication during informed consent. We are contacting them now and discussing the availability of the data.

(2) p. 6, please define what is a sizeable correlation: provide an actual number. You can even drop the term sizeable, the number can speak for itself.

Response: We added that number as:

“We considered that the sample size was relatively small but sufficient for this study, because a minimum sample size of 84 is required to achieve “sizeable” correlation coefficients (>0.3) with a power of 0.8 (Bujang & Baharum, 2016).”

(3) p. 9, please define what a true negative rate is.

Response: We revised the corresponding sentence as:

“…and mean true negative rate (i.e., rate of correct [no-go] response to the target) was 0.71 (SD: 0.17, range: 0.17­­–1).”

(4) p.9, please list the reliability of each scale in Table 1.

Response: We added this information to Table 1.

(5) Similarly to Reviewer 1, I think you have a rich data-set with the SART, and I would like to see a detailed assessment of how performance in the SART relate to self-reported mind-wandering. One can evaluate, for example, whether mean and variability in RTs predict MW and accuracy in no-go trials. If probes followed no-go trials, one can assess whether no-go accuracy is associated with reported MW.

Response: As with the response to 5) of reviewer 2, the effect of task performance was not strong enough to affect the correlation of state level motivation-MW association. We would have to set rigorous categorization of MW to assess the correlation between task performance and MW rates because the self-report of MW often includes task-related thoughts, as one of the authors reported (Kawagoe & Kase, 2020, *Psychological Research*).

(6) At moments, I felt the paper had too many abbreviations. Consider using words instead of abbreviations because translating their meaning creates a burden to the reader.

Response: Although we have tried to reduce them, the abbreviations used here were only indices of each variables except the word of MW for mind wandering. Using full words for those indices seems to be uncomfortable to read (e.g., association between mind wandering questionnaire and day dreaming frequency scale was…).

We truly thank all of the reviewers for their valuable comments and believe that the manuscript has been significantly improved as a result. However, we are willing to revise our manuscript again if further revisions are considered necessary.

Yours sincerely,

Toshikazu Kawagoe

College of Contemporary Psychology, Rikkyo University
